# Potential Effects of Coronaviruses on the Liver: An Update

**DOI:** 10.3389/fmed.2021.651658

**Published:** 2021-09-27

**Authors:** Xinyi Wang, Jianyong Lei, Zhihui Li, Lunan Yan

**Affiliations:** ^1^Thyroid and Parathyroid Surgery Center, West China Hospital of Sichuan University, Chengdu, China; ^2^Liver Surgery Center, West China Hospital of Sichuan University, Chengdu, China

**Keywords:** coronavirus, COVID-19, liver injury, liver diseases, drug hepatotoxicity

## Abstract

The coronaviruses that cause notable diseases, namely, severe acute respiratory syndrome (SARS), middle east respiratory syndrome (MERS) and coronavirus disease 2019 (COVID-19), exhibit remarkable similarities in genomic components and pathogenetic mechanisms. Although coronaviruses have widely been studied as respiratory tract pathogens, their effects on the hepatobiliary system have seldom been reported. Overall, the manifestations of liver injury caused by coronaviruses typically involve decreased albumin and elevated aminotransferase and bilirubin levels. Several pathophysiological hypotheses have been proposed, including direct damage, immune-mediated injury, ischemia and hypoxia, thrombosis and drug hepatotoxicity. The interaction between pre-existing liver disease and coronavirus infection has been illustrated, whereby coronaviruses influence the occurrence, severity, prognosis and treatment of liver diseases. Drugs and vaccines used for treating and preventing coronavirus infection also have hepatotoxicity. Currently, the establishment of optimized therapy for coronavirus infection and liver disease comorbidity is of significance, warranting further safety tests, animal trials and clinical trials.

## Introduction

Coronavirus (CoV) is a family of viruses that display crown-like structures under electron microscopy, with an outer envelope and positive-stranded RNA as the genomic material (1). These viruses are found widely in many species, including humans, mice, pigs and other animals (2, 3). To date, 7 types of coronaviruses have been shown to cause disease in humans, of which 4 species (alpha CoVs: HCoV-NL63, HCoV-229E; beta CoVs: HCoV-OC43, HCoV-HKU1) can cause self-limiting respiratory symptoms in immunocompromised people, infants and older individuals (4). Another three species (SARS-CoV, MERS-CoV, and SARS-CoV-2) are highly pathogenic to humans, causing respiratory diseases, and the infection may lead to acute respiratory distress syndrome (ARDS), multiple organ failure (MOF) and even death in severe cases (5, 6). As coronaviruses have been widely studied as human respiratory pathogens, their involvement in the hepatobiliary systems needs further investigation.

The occurrence of recent coronavirus outbreaks has revealed that these viruses can mutate to become pathogenic in both humans and animals (7). As virus variations are inevitable and a part of the evolutionary process, outbreaks of coronaviruses will continue to emerge (8). SARS-CoV was the first causative agent of human pathogenic coronavirus outbreak globally, occurring in Guangdong Province of China in 2002–2003 (9), and it can cause severe respiratory syndrome with mortality rate of 9% (7). During this outbreak, ~8,098 human cases of SARS were reported, and 774 of these patients died of the infection (10). The next coronavirus outbreak that followed the SARS-CoV outbreak was the MERS-CoV outbreak (11). Occurring in 2012, this outbreak involved severe infections in the respiratory tract of infected individuals in Saudi Arabia and other Middle East countries (12). The initial mortality rate of MERS-CoV was ~50%, but the outbreak was over by 2013, with only a few sporadic cases since (13). Based on the latest update from the WHO, the total number of reported cases of MERS-CoV worldwide was 2,519,866 of the patients died, resulting in a mortality rate of 34.4% (14). The most recent coronavirus outbreak occurred in Wuhan, China, which was also known as the 2019-nCoV outbreak; the virus was recently renamed SARS-CoV-2, and the disease is referred to as COVID-19 (15). The first case of SARS-CoV-2 infection was reported in Wuhan, China, on 31 December 2019 with symptoms of atypical pneumonia (16). This case was later proven to be caused by a novel coronavirus, SARS-CoV-2. According to the WHO, as of 10 AM CET 2nd July 2021, 187,882,032 cases of COVID-19 have been reported, with 4,046,592 deaths, worldwide (17). There were 34,766,404 confirmed cases of SARS-CoV-2 infections in the USA, including 623,039 deaths. In terms of death related to COVID-19, after the USA, the greatest number of deaths due to COVID-19 has been reported in Brazil (534,233), followed by India (408,764).

Despite remarkably high genetic similarity between SARS-CoV and SARS-CoV-2 with regard to gene sequence, the speed at which SARS-CoV-2 spreads is much faster than that of SARS-CoV (18). This may be explained by differences in the structure of spike proteins (S proteins) among coronaviruses (19). The S protein is a 150-kDa protein that is highly N-glycosylated and plays roles in interaction with the endoplasmic reticulum (ER) and receptor attachment (20, 21). Usually, the S protein is cleaved into two functional domains (S1 and S2) by a host protease (furin-like protease) (22, 23). The presence of this special protease cleavage site activates S protein the priming and might improve the efficiency of SARS-CoV-2 transmission (24). The S protein also serves as a ligand on the coronavirus surface, which binds to angiotensin-converting enzyme 2 (ACE-2) (25). SARS-CoV and SARS-CoV-2 use the ACE2 receptor of the host cell, whereas MERS-CoV binds to dipeptidyl-peptidase 4 (DPP4) (26–29). After attaching to the cell membrane, the viral genome enters the cytoplasm and is translated to produce new virions, which can further lead to infection and respiratory disease (30, 31). This mechanism has become the most likely reason for multiple organ dysfunction in patients with coronavirus infection (31, 32).

### Liver Injury in Patients With Coronavirus Infection

#### Manifestations of Coronavirus-Related Liver Injury

Coronavirus infections are distinguished by continuous fever, cough, fatigue, dyspnea, arthralgias and decreased white blood cells in the serum (13, 33, 34). The severity of coronavirus infection is evaluated by the degree of respiratory symptoms and intensive care unit (ICU) admission (35, 36). It is notable that coronaviruses can influence not only the respiratory system but also the digestive, cardiac and endocrine systems (37, 38). Indeed, one study found that diarrhea occurred in 3.8% of COVID-19 patients and that 43.4% of patients had different degrees of liver function abnormality (39). Moreover, the incidence of liver injury in severe COVID-19 cases (74.4%) was higher than that of patients with mild disease (43.0%) (40). In cases of death due to COVID-19, the incidence of liver injury is 58% (40). According to the autopsy report of SARS patients, many virus particles were observed in the lung and the parenchymal areas and vascular endothelium of other organs, such as the liver (41, 42). The genome of SARS-CoV was also detected in liver tissue by RT-PCR (43). Among the three notable coronaviruses, acute liver injury has been mostly reported in MERS-CoV infection (44), and according to a study from Saad et al., 31.4% of patients have liver dysfunction during MERS-CoV infection (45). The common manifestations of liver injury caused by infections of the three coronaviruses are summarized in Table 1.

**Table 1:** The manifestations of coronavirus-induced liver injury.

	**SARS-CoV-2**	**SARS-CoV**	**MERS-CoV**
Pathological changes	Mild lobular infiltration by small lymphocytes, centrilobular sinusoidal dilation and patchy necrosis	Accumulation of cells in mitosis, ballooning of hepatocytes and mild lobular lymphocytic infiltration.	Moderate portal tract infection, lobular lymphocytic inflammation and hydropic degeneration of hepatic parenchymal cell
	ref: Tian et al. (46)	ref: Chau et al. (47)	ref: Ng et al. (48)
ALT	Elevated (affected proportion: 13.3–28.0%)	Elevated (affected proportion: 52.5–87.0%)	Elevated (affected proportion: 11.0–56.3%)
	ref: Guan et al. (33), Chen et al. (49)	ref: Jiang et al. (50), Liu et al. (51)	ref: Arabi et al. (52)
AST	Elevated (affected proportion: 22.0–58.0%)	Elevated (affected proportion: 37.1–86.9%)	Elevated (affected proportion: 15.0–86.8%)
	ref: Guan et al. (33), Chen et al. (49)	ref: Jiang et al. (50), Liu et al. (51)	ref: Arabi et al. (52)
TB	Elevated (affected proportion: 10.5–18.0%)	Elevated (affected proportion: 30.0%)	Not available
	ref: Guan et al. (33), Chen et al. (49)	ref: Jiang et al. (50)	
Albumin	Decreased (affected proportion: 36.8%)	Decreased (affected proportion: 40.4–72.0%)	Not available
	ref: Zhang et al. (53)	ref: Jiang et al. (50)	
Comorbidity with liver disease	The proportion of severe cases in patients with HBV comorbidity is higher than that of patients without HBV infection. (32.9 vs. 15.3%)	Chronic hepatitis B was not associated with worse clinical outcomes.	Not available
	ref: Wu. et al. (54)	ref: Huang et al. (55)	

*ALT, glutamic-pyruvic transaminase; AST, glutamic-oxalacetic transaminease; TB, total bilirubin*.

The latest studies on SARS-CoV-2 have indicated that the incidence of liver injury in patients with COVID-19 ranges from 14.8 to 53%, manifesting as abnormal glutamic-pyruvic transaminase (ALT), glutamic-oxalacetic transaminase (AST) and bilirubin levels (33, 53, 56). Moreover, compared with mild COVID-19 cases, severe cases show higher levels of plasma ALT and AST (57). The risk of being transferred to the ICU and critical care unit (CCU) is statistically correlated with elevated AST and bilirubin levels, and mortality correlates positively with elevated AST levels (58). Injury to bile duct cells and abnormal gamma-glutamyl transferase (GGT) and alkaline phosphatase (ALP) levels have also been found in COVID-19 patients (57, 59, 60). This is a transient reaction, and therefore, the ALT levels of most patients usually return to normal after recovery (61). Patients with persistent high ALT level were in severe condition or has basic liver diseases, being found to have higher rates of 30-day mortality and longer hospitalization (62). Albumin is decreased in severe cases (~26.3–30.9 g/L) and correlates with disease severity and mortality (36, 57, 63). Low levels of prealbumin in severe SARS-CoV-2 patients have also been reported, suggesting that hepatic synthesis is suppressed in these patients (53). Similarly, liver injury in SARS and MERS patients is characterized by mild increases in ALT, AST and bilirubin at the early stage of the disease (35, 44, 50, 51, 64–67). Moreover, great elevation of liver enzymes is an independent factor correlating with a poor prognosis of patients with SARS (68). Although age and pre-existing diseases have been proven to have a significant negative influence on the prognosis of SARS patients, patient age was not significantly different between those with high or low peak ALT levels (69, 70). In a cohort of severe MERS patients, 50% exhibited elevated aminotransferase levels during their time in the ICU (52, 71, 72). Saad et al. illustrated that decreased albumin level is a predictive factor of the severity of MERS (73).

**Figure 1:**
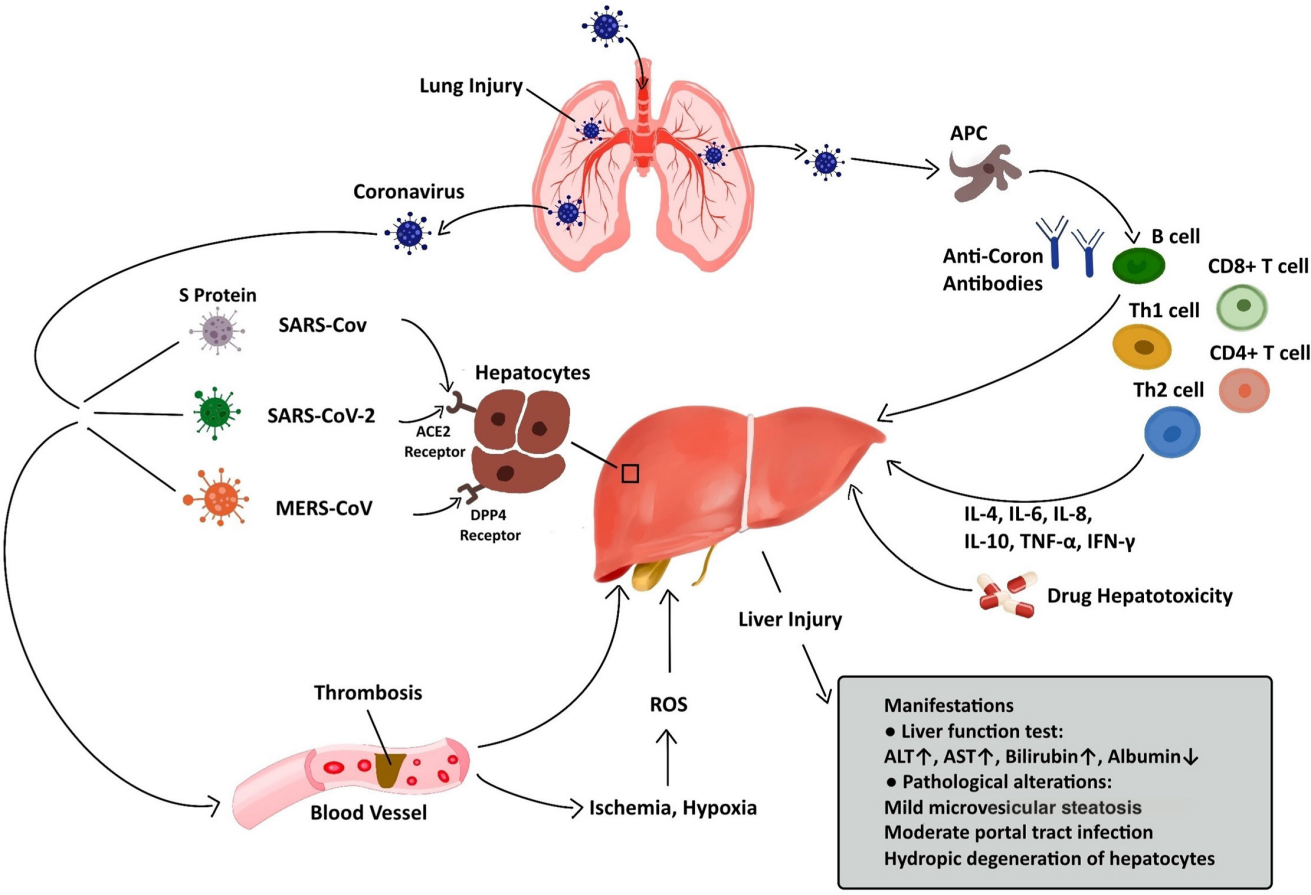
Liver injury caused by coronavirus infection. After entry into the human body through the respiratory tract, coronaviruses can lead to liver injury via several approaches, including ACE2/DPP4-mediated hepatocyte injury, immune-mediated liver injury, ischemia and hypoxia, thrombosis, and drug hepatotoxicity. The manifestations of liver injury involve abnormalities in liver function test and pathological examination.

Regarding pathological changes, liver biopsies of SARS patients revealed dramatic increases in eosinophilic bodies and balloon-like hepatocytes, indicating that coronavirus might cause necrosis of hepatocytes (47). Some studies showed that protein 7a, a special protein of SARS-CoV, can also induce necrosis of cell lines belonging to various organs (74, 75). Mild microvascular steatosis and moderate lobular and portal inflammation have been found in the livers of patients with SARS-CoV-2 infection (46). Similar to the observations for SARS and COVID-19 patients, the pathological changes in MERS patients include moderate portal tract infection, lobular lymphocytic inflammation, and hydropic degeneration of hepatic parenchymal cells (48, 76). The definitive mechanism by which liver injury develops in patients with coronavirus infection is unclear, and several pathophysiological theories may explain this phenomenon (Figure 1, Table 2).

**Table 2:** The mechanisms of coronavirus-induced liver injury.

**Pathogenic mechanism**	**Coronavirus type**	**References**	**Highlights**
ACE2/DPP4-mediated direct injury of hepatocytes	SARS-CoV	Li et al. (27)	ACE2 was shown to be the functional receptor of SARS-CoV. ACE2 will likely contribute to the development of antivirals and vaccines.
	SARS-CoV-2	Bourgonje et al. (28)	ACE2 has been established as the functional host receptor for SARS-CoV-2. ACE2 expression and activity are related to COVID-19 severity. ACE2 inhibitor is a selection of potential treatment modalities for COVID-19.
	MERS-CoV	Wang et al. (29)	The receptor-binding subdomain is critical for viral binding to DPP4 and entry into the target cell.
Immune-mediated injury	SARS-CoV	Duan et al. (68)	IL-1, IL-6, and IL-10 in the serum of SARS patients with abnormal liver function were higher than those in patients with normal liver function.
	MERS-CoV	Mahallawi et al. (77)	IFN-γ, TNF-α, IL-15, and IL-17 were significantly increased in those infected by MERS-CoV.
	SARS-CoV-2	Huang et al. (38)	TNF-α, IFN-γ, IL-6, IL-8, IL-4, and IL-10 were dramatically elevated in COVID-19 patients.
Thrombosis	SARS-CoV, SARS-CoV-2, and MERS-CoV	Giannis et al. (78)	There was a great proportion of patients with hypercoagulable states after coronavirus infection.
	SARS-CoV-2	Llitjos et al. (79)	Elevated D-dimer level and thrombocytopenia were observed in some COVID-19 patients.
Drug hepatotoxicity	SARS-CoV, SARS-CoV-2, MERS-CoV	Sheahan et al. (80)	The anti-corona acitivities of remdesivir has been reported. Redesivir can cause elevation in aminotransferases.
	SARS-CoV	Cao et al. (81)	Lopinavir/Ritonavir can cause elevation in serum amylase and liver enzymes.
	SARS-CoV-2	Fan et al. (39)	A significantly higher proportion of patients with abnormal liver function had received lopinavir/ritonavir after admission.
	SARS-CoV-2	Xu et al. (82)	Tocilizumabcan cause mild elevation in serum aminotransferase, jaundice and occasional reactivation of hepatitis B.

*ACE2, angiotensin-converting enzyme 2; DPP-4, dipeptidyl-peptidase 4*.

#### Pathogenic Mechanisms of Coronavirus-Related Liver Injury

##### ACE2/DPP4-Mediated Damage to Hepatocytes

RAS proteins are encoded by Ras sarcoma oncogenes and belong to a group of small GDP/GTP-binding guanine triphosphatases, which play an essential role in cellular biological behaviors such as proliferation, migration, adhesion, and differentiation (83). Abnormal signaling of RAS occurs in numerous human diseases (84). ACE2 plays an important role in the RAS signaling pathway by upregulating angiotensin II (Ang II), which promotes atherosclerosis, inflammation, and migration of endothelial cells (85). ACE2 is widely present in humans, including in alveolar epithelium, intestinal epithelium and arterial smooth muscle cells (86). Furthermore, it has been confirmed that ACE2 receptors are over-expressed in gastrointestinal epithelium enabling viruses to invade bile duct cells and suppress liver function (40, 86). Herath et al. reported both liver tissue and bile duct epithelium express ACE2 (87). However, the ACE2 expression level in bile duct epithelium was significantly higher than that in liver tissue (88). As bile duct cells play essential roles in hepatic regeneration and the immune activities, upregulation of ACE2 expression in hepatocytes can lead to compensatory proliferation originating from bile duct cells, resulting in liver injury (88, 89). Although SARS-CoV and SARS-CoV-2 can cause liver function abnormality through binding to the ACE2 receptors of bile duct cells, viral inclusions were not observed in the liver biopsies of COVID-19 patients (46). These results indicate that liver injury in patients with coronavirus infection may be the result of bile duct epithelium damage rather than hepatocyte changes (90). Numerous literature have reported liver cirrhosis can also dramatically upregulate ACE2 expression in hepatocytes (91–93). In the normal human liver, ACE2 stains weakly and is limited to the bile duct cells, vascular endothelial cells, and perivenular hepatocytes (86, 87). In the cirrhotic liver, ACE2 staining can be observed in the majority of hepatocytes in the cirrhotic nodules, bile duct and vascular endothelium (88). High expression of ACE2 helps more coronaviruses invade hepatocytes and leads to greater virulence of coronaviruses in the liver (87, 94, 95). Thus, patients with both liver cirrhosis and coronavirus infection may have greater extents of liver dysfunction and even higher risks of liver failure compared with normal people (96, 97).

Dipeptidyl peptidase 4 (DPP-4) cleaves a large number of chemokine and peptide hormones involved in the regulation of the immune system (98). DPP-4 is upregulated in the liver, indicating that the liver might be a target organ of MERS-CoV (99, 100). A scientific team built a transgenic murine model which expressed codon-optimized human DPP-4 (hDPP-4) and observed that MERS-CoV can invade into the hepatocytes through DPP-4 and cause hepatocytes injury (101). The hDPP-4 transgenic mouse exhibited mild hepatic injury on the 5th day after MERS-CoV infection, and the pathological manifestations were scattered necrosis of hepatocytes in sinuses and infiltration of numerous macrophages and Kupffer cells (102, 103). On the 9th day, although hepatocytes necrosis was less, fatty changes in hepatocytes were also found (104).

##### Immune-Mediated Injury

When coronaviruses invade the human body, they activate the immune system, triggering a series of immune activities to eliminate the virus (105–107). The liver plays an essential role in immune activities and contains numerous immune cells that participate in the immune response (108–110). The hepatic acute-phase response (involving cytokines released from immune cells) is a defense reaction to fight against the pathogen and protect vital liver functions (111, 112). T cells play important roles in the anti-coronavirus immune responses, and the balance between the anti-coronavirus response and immune tolerance is maintained by the differentiation of CD4+ and CD8+ T cells (113). During the process of SARS-CoV-2 infection, 80% of immune cells that infiltrate into the liver are CD8+ T cell, and these cells could survive in the inflamed tissue (114). The decrease in the infiltration of CD4+ T cell can lead to depressed B cell activation, along with reduced level of SARS-CoV-2-specific neutralizing antibody and pro-inflammatory cytokine (such as IL-1, IL-6, and TNF-α), so as to affect the clearance of SARS-CoV-2 from the liver (115). Compared with SARS-CoV-2, CD4+ T cell is more susceptible than CD8+ cell during the processes of MERS-CoV and SARS-CoV infections. Liver cells in patients with severe coronavirus infection show various inflammatory changes, such as swelling and steatosis in hepatocytes, proliferation in liver sinus cells, hyperplasia in Kupffer cells and infiltration in immune cells (40, 46, 116). Cytokines can also induce ischemia and hypoxia, which lead to hepatocyte injury and necrosis (117).

Abnormal serum levels of cytokines and chemokines (such as tumor necrosis factor (TNF), interleukin-6 (IL-6), and IL-18) have been detected at the early stage of coronavirus infection (111, 118). Duan et al. found that the concentrations of IL-1, IL-6, and IL-10 in the blood of SARS patients with hepatic dysfunction were higher than those in patients with normal hepatic function, demonstrating the relevance between hepatic injury and the cytokine storms caused by SARS (68). The levels of IL-2-receptor and IL-6 in the serum of patients with SARS-CoV-2 infection were also be found to be elevated and relate to the disease severity (119). Moreover, cytokines secreted by Th1 and Th2 cells (involving TNF-α, IFN-γ, IL-6, IL-8, IL-4, and IL-10), are dramatically elevated in patients with SARS-CoV-2 infection [38]. During the acute phase of MERS-CoV infection, the levels of IFN-γ, TNF-α, IL-15, and IL-17 in the serum of patients were dramatically elevated (77). These results suggest that the systemic inflammatory reaction syndrome (SIRS) and cytokine storms caused by coronavirus infection may be critical mechanisms of liver injury (68, 120, 121). Nonetheless, there is a lack of research on the relationship between pro-inflammatory cytokine activity and liver injury.

##### Ischemia and Hypoxia

Patients with SARS-CoV-2 infection exhibit different extents of hypoxemia, with more than 40% of patients receiving oxygen treatment (117). Hypoxic liver injury can be marked by increased transaminases in the serum due to dysregulation of the oxygen supply (122). Complications of COVID-19, including ARDS, SIRS and MOF, can lead to hypoxemia, ischemia and shock (123, 124), and microthrombi can disrupt perfusion within the liver. Hepatic sinus endothelial cells also play roles in the occurrence of this phenomenon, as they can respond to inflammatory signals (such as endotoxins with endothelium dysfunction, characterized by reduced vasodilatory responds to acetylcholine and reduced nitric oxide synthase phosphorylation). Hepatic ischemia–reperfusion injury (HIRI) is another familiar pathological process, whose mechanism is closely correlated with reactive oxygen species (ROS), neutrophils, Kupffer cells, and overloaded calcium. HIRI can lead to inflammation and cell injury by activating Kupffer cells, neutrophils, and platelets. Under the circumstance of ischemia and hypoxia, the cell survival signaling pathway in hepatocytes can be inhibited by glycogen consumption and adenosine triphosphate depletion, resulting in necrosis of these cells (125). Moreover, for patients who develop ARDS, hypoxia can cause oxidative stress responses that facilitate a persistent elevation of ROS (126). ROS and their per-oxidized forms can arouse regulation of redox reactions and promote the secretion of pro-inflammatory substances to cause hepatic injury (127, 128). These pathophysiological changes may accentuate liver ischemia and hypoxia, influencing the secretion of hepatotoxic substances and so as to affect hepatic function (125, 129).

##### Thrombosis

SARS-CoV, MERS-CoV, and SARS-CoV-2 have been reported to lead to hypercoagulable states in patients, thus increasing the chance of thrombosis (78). Previous studies on COVID-19 have shown that 36.2% of patients developed thrombocytopenia, 46.4% of patients had increased D-dimer levels during infection, and the rates were higher in severe than in mild cases (79). It has recently been reported that microvascular thrombosis can lead to end-stage organ injury and can potentially influence hepatic function (130, 131). In the past, elevated levels of serum ALP was considered as a prognostic factor for ischemic stroke and a risk factor for hemorrhagic transformation (132). COVID-19 patients who experienced thrombotic events had dramatically high levels of ALP, though ALP levels were normal or only mildly increased in patients without thrombotic events (133). Recent data suggest that COVID-19 patients have a greater chance of developing disseminated intravascular coagulation (134, 135). Elevated D-dimer levels, the level of degradation products of fibrin, and prolonged prothrombin time have also been shown to be correlated with worse prognosis of patients with SARS-CoV-2 (136). The results of autopsies from Wuhan have revealed lymphocytes and monocytes infiltration in the portal area, with thrombosis and congestion in the sinuses (116). The liver was found to have hepatocyte degeneration along with lobular necrosis and neutrophil infiltration (46, 116). These findings suggest that hypercoagulable states in patients with COVID-19 are a potential reason for liver injury.

##### Drug Hepatotoxicity

Drug hepatotoxicity is the third leading cause of liver injury after viral hepatitis and alcoholic/non-alcoholic fatty liver disease (137). Based on many clinical studies and animal experiments, several types of drugs have been proven to cause liver injury, including antibiotics, anti-tumor drugs, saikosaponins, anti-tuberculosis drugs, and anti-malarial drugs (138–140). Until now, there are no effective therapeutic treatments for patients with SARS (141, 142). Drugs that were mostly chosen for SARS patients were ribavirin and corticosteroids (143). Ribavirin was used because it had a broad spectrum of activity against RNA viruses, and steroids were chosen because of their anti-inflammatory functions (144, 145). Nevertheless, ribavirin is correlated with obvious hepatotoxicity, including hemolysis, resulting from discontinuation of its use (143). Most patients with SARS-CoV-2 infection have fever and take antipyretic drugs that contain acetaminophen (38, 146). Acetaminophen is known to lead to liver injury, and acetaminophen overdose can induce serious liver injury or even liver failure (147). COVID-19 patients have been treated with lopinavir, abidor, ritonavir, and other antiviral drugs (146). Furthermore, some scientists proposed that HIV protease inhibitors might efficiently inhibit the replication of SARS-CoV-2 (148, 149). However, Shen et al. proved the chance of having liver injury was increased in patients who received both hormone therapy and HIV protease inhibitors (150, 151). Intravenous methylprednisolone was also reported to correlate with acute liver injury, but evidence on the correlation between oral methylprednisolone and liver injury is insufficient (152). We have summarized the effects of several anti-corona drugs on liver function in Table 3. Some clinical trials on anti-SARS-CoV-2 drugs are still ongoing (Table 4).

**Table 3:** Drug-induced abnormal liver function in patients with coronaviruses infection.

**Drugs for coronaviruses infection**	**Number of cases**	**Proportion of liver injury**	**ALT**	**AST**	**ALP**	**Total bilirubin**	**γ-glutamyltransferase**	**References**
Remdesivir	387	34.0% (130/387)	20.4% (79/387)	20.4% (79/387)	Not available	1% (4/387)	Not available	(153)
Lopinavir/ritonavir	148	37.2% (55/148)	18.2% (27/148)	21.6% (32/148)	4.1% (6/148)	6.1% (9/148)	17.6% (26/148)	(39)
Interferon	31	38.7% (12/31)	38.7% (12/31)	29.0% (9/31)	32.3% (10/31)	16.1% (5/31)	Not available	(154)
Baricitinib	12	58.3% (7/12)	58.3% (7/12)	50.0% (6/12)	Not available	Not available	Not available	(155)
Tocilizumab	20	5.0% (1/20)	5.0% (1/20)	5.0% (1/20)	Not available	Not available	Not available	(82)

*ALT, glutamic-pyruvic transaminase; AST, glutamic-oxalacetic transaminease; ALP, alkaline phosphatase*.

**Table 4:** The efficacy and current status of COVID drugs.

**Drugs**	**Mechanisms**	**Efficacy**	**Clinical trials**
Remdesivir	Inhibiting viral replication by interfering RNA polymerases	To be tested in clinical trials	NCT04292899 NCT04257656 NCT04292730
Lopinavir/Ritonavir	Inhibiting viral replication by interfering protease	Inconsistent results in completed clinical trials	NCT04343768 ChiCTR2000029308
Interferons	Directly inhibit viral replication and transmission and support immune responses to clear viruses.	To be tested in clinical trials	NCT04389645 NCT04276688
Baricitinib	JAK inhibitors	Proven efficacy (baricitinib plus remdesivir)	NCT04401579
Tocilizumab	Humanized mAb targeting IL-6	Do not improve survival	NCT04372186
rhACE2	Completely bind to viral S-protein	To be tested in clinical trials	Not available

## Effects of Coronavirus Infection on Pre-Existing Liver Disease

Chronic liver disease is one of the biggest disease burdens, accounting for about 1 million deaths per year worldwide (156–158). As a result, the influence of coronaviruses on various pre-existing liver diseases needs to be further explored; evidence of active viral replication and persistent liver injury after coronavirus infection also calls for further investigation (159). For patients with pre-existing liver diseases, the addition of coronavirus-directed or immune-response-directed liver injury may lead to further hepatic dysfunction, especially for patients with advanced liver diseases. As an example, experience obtained from the SARS pandemic in 2003 showed that comorbidity with hepatitis B can cause more severe liver injury (160). However, if the liver injury caused by COVID-19 is immune-response-directed, the immunocompromised condition of cirrhosis patients and cancer patients may be more beneficial than detrimental (161). Moreover, patients with liver cirrhosis or liver cancer are usually in an immunocompromised state and may be more susceptible to SARS-CoV-2 infection (162, 163). Clinical practice guidance regarding liver disease has been given to healthcare professionals by relevant societies worldwide, including the American Society of Clinical Oncology (ASCO), the European Society for Medical Oncology (ESMO), the International Liver Cancer Association (ILCA), the European Association for the Study of the Liver (EASL), and the American Association for the Study of Liver Diseases (AASLD) (164–169). Here, we summarize the effect of coronavirus infections on the occurrence, development and treatment of four types of liver diseases: viral hepatitis, liver cirrhosis, hepatocellular carcinoma and liver transplantation.

### Effect of Coronaviruses on HBV and HCV Hepatitis

HBV and HCV are chronic infections that occur frequently worldwide, with 2 billion people infected and 350 million having chronic infection (170, 171). One study indicated that 3.6 and 0.6% of patients with COVID-19 had a history of hepatitis B and hepatitis C, respectively (172). In a study about hepatic biochemical parameters in 324 cases in Shanghai, the percentage of COVID-19 patients with HBsAg positivity was 6.5% (173). Thus, the influence of coronavirus infection on the course of HBV and HCV has attracted widespread attention (174, 175). SARS patients with HBV and/or HCV infection had a higher risk to get liver injury and severe hepatitis because hepatitis virus replication was promoted during SARS-CoV coinfection (55, 67). However, considering coinfection of SARS-CoV, no significant differences in various adverse clinical outcomes between chronic hepatitis B patients and HBsAg-negative patients were detected (176). SARS patients with acute hepatitis and/or decompensated liver cirrhosis have a greater chance to be dead (47). A research team reported that 23/1099 SARS-CoV-2 patients in Wuhan were coinfected by HBV, representing 2.4% of mild cases and 0.6% of severe cases (174). COVID-19 patients also had a higher mortality rate than that of HBV-negative patients (32.9 vs. 15.3%) (54). Liu et al. found that the median time of virus clearance (21 days, 95% CI: 19–29) in COVID-19 patients with HBV infection was longer than that in patients without HBV infection (14 days, 95%, CI: 13–21) (177). These results indicate that coronavirus infection and viral hepatitis interact; thus, exploring the underlying mechanism will be meaningful for optimizing treatment guidance for COVID-19.

The presence of coronavirus infection and complications should be factors considered when doctors develop tailored treatment plans for patients with HBV and/or HCV infection (178). According to the AASLD guidance, we should initiate anti-HBV/HCV therapy in patients under three states: 1) newly diagnosed cases of HBV/HCV; 2) patients without SARS-CoV-2 infections; 3) if resources (involving drug treatments, personnel for approval of therapy, blood testing, follow-up facilities through telemedicine or face-to-face) have not been deployed for SARS-CoV-2 infection (168). HBV reactivations after using tocilizumab or prednisone have been reported in patients with HBV infection; therefore, these two drugs should not be used to avoid HBV reactivation (179). Additionally, according to guidance from AASLD, long-term HBV therapy can be employed for patients with newly diagnosed HBV hepatitis and continued if the patients receive the therapy plan, regardless of whether the patients are infected by SARS-CoV-2 (168). Therefore, therapy guidance for COVID-19 patients with advanced liver disease needs to be established to minimize the risk of liver injury or even liver failure, as both the advantages and disadvantages of an intervention are vital during the treatment of COVID-19.

For hepatitis B patients who are undergoing antiviral treatment and high-dose hormone therapy, discontinuation of anti-HBV therapy might cause reactivation and replication of HBV during SARS-CoV-2 infection (180). Indeed, studies have pointed out that treating HBV/HCV patients with lopinavir and ritonavir can increase the incidence of liver injury (181–183). A clinical study showed that long-term application of ribavirin can lead to serious drug hepatotoxicity in HCV patients, which may be due to metabolic reactions in the body (184). In addition, patients with HBsAg positivity and hepatitis B core antibody positivity treated with corticosteroids showed a higher risk of HBV reactivation, and the incidence of HBV reactivation correlates with the dosage of corticosteroid treatment (177, 180). Therefore, the clinical status of chronic HBV infection should be systematically evaluated in the setting of corticosteroid use, and nucleotide analog treatment should be taken into consideration to reduce the risk of HBV reactivation or hepatitis flare.

### Effect of Coronaviruses on Liver Cirrhosis

It is known that liver cirrhosis is one of the leading causes of death and illness globally; thus, exploring how coronavirus infection influences the course of liver cirrhosis is of great importance (162, 185). For patients with liver cirrhosis and coronavirus infection, the severity of COVID-19 and the incidence of severe complications increase, resulting in a higher liver-related mortality rate compared to patients with COVID-19 alone (49). A clinical study demonstrated that SARS-CoV-2 infection can lead to rapid deterioration in patients with relatively stable liver cirrhosis: 25 COVID-19 patients with Child-Pugh A presented rapid deterioration in hepatic function, and the Child-Pugh scores of over 30% of them increased to B or C after COVID-19 diagnosis (96). The last hospital admission or follow-up visit before COVID-19 diagnosis provides evidence for the importance of SARS-CoV-2 infection in deteriorating hepatic function, which can usually be seen in patients with liver cirrhosis of any etiology (186). However, more evidence is needed to completely clarify the effect of elevated ALT on the disease course of patients with liver cirrhosis and COVID-19 and to further explain the pathogenic mechanism by which coronavirus induces hepatocyte injury (57). The potential cytopathic effect has been demonstrated, as numerous ACE2 receptors might help SARS-CoV-2 enter host liver cells (32). Alternatively, the liver might be indirectly involved in acute inflammatory activity after SARS-CoV-2 infection, as it becomes infiltrated with a large number of macrophages, potential cytokine producers (107).

To date, drugs that have been widely used for COVID-19 treatment included chloroquine, lopinavir/ritonavir, ribavirin, favipiravir, remdesivir, and tocilizumab et al. (146, 187). As the majority of these drugs are metabolized in the liver, abnormal hepatic function might increase the risk of drug hepatotoxicity in COVID-19 patients (188). It is worth noting patients with pre-existing liver diseases, especially liver cirrhosis with Child-Pugh B/C, have a greater chance of experiencing adverse reactions to the above drugs (189). As a result, close and frequent monitoring of hepatic bio-parameters in patients can help in the notification of liver injury and reduce the risk of adverse effects and optimize drug dosages (137). It is recognized that endoscopic variceal screening in healthy individuals should be restricted to people with high risk for variceal bleeding, as well as those with histories of variceal bleeding or portal hypertension (190); otherwise, non-invasive examinations for the diagnosis should be performed (191). To decrease the risk of spreading infection, endoscopy examination in COVID-19 patients need to be restricted to critical situations such as gastrointestinal bleeding.

### Effect of Coronaviruses on Liver Cancers

Patients with liver cancers also have a high risk of coronavirus infection, especially if they receive chemotherapy or immunotherapy in the hospital (192). The incidence of COVID-19 in cancer patients at a hospital in Wuhan was 0.79% (12/1,524), higher than that of the whole community during the same period (193). Owing to the serous spread of the COVID-19 pandemic, <50% of them were being continuously treated for their cancer (194). Furthermore, cancer patients have poorer prognosis than patients with COVID-19 alone, with the mortality rate ranging from 5 to 20% (195).

Patients with liver cancer should accept special treatments and take interventions to prevent severe complications of COVID-19 (196). In patient-saturated hospitals, the shortage of clinical and medical resources has largely impeded normal radiological examination, pathological diagnosis and anticancer treatment for patients with liver cancer (197). EASL, ESMO, and ILCA have provided specific guidance for the surveillance, examination and treatment of liver cancer patients with SARS-CoV-2 infection (165, 167, 169, 198). According to the Society of Surgical Oncology, all patients with aggressive liver, pancreatic or gall bladder cancers should undergo surgery (199). For patients who need surgery as well as systemic chemotherapy, neoadjuvant chemotherapy should be considered to delay the surgery.

Screening for esophageal varices and liver cancers is now delayed for all but high-risk patients (186). AASLD guidance suggests that it is appropriate to delay liver cancer surveillance for 2 months after evaluation of the advantages and disadvantages of initiating liver cancer surveillance in COVID-19 patients. Some retrospective studies have indicated that semiannual surveillance can increase the possibility of early detection and improve patients' survival compared with annual surveillance (200). Therefore, delaying screening for over 1 year may lead to progression of liver cancer, resulting in the miss of the best time for operating and even liver failure or death. Delaying HCC surveillance over short periods of time is likely acceptable as the annual HCC incidence is 2–3%, meaning that 98% of people will not develop HCC in a surveillance interval (201). These changes in treatments have potentially increased the risk for variceal bleeding and distant metastasis of liver cancer. Additionally, living donor liver transplantation and locoregional therapy for liver cancers have been delayed in many institutions, possibly increasing both the progression and mortality of liver cancer (168). Selective strategies included using serum biomarkers, increasing outpatient interventions (such as albumin infusions), and integrated telehealth are being strongly recommended by many institutions (202).

### Effect of Coronaviruses on Liver Transplantation

After liver transplantation, patients have a greater chance to be infected and/or get severe course of COVID-19 because of their immunosuppressed state (203). These patients are being treated with immunosuppressive drugs and are considered to have greater chances of contracting SARS-CoV-2 infection, resulting in serious complications (1.4% death, 5.0% admitted to the ICU and 15.7% severe disease) (204). Gwilym et al. performed a study involving 151 liver transplant recipients, and reported that previous liver transplantation does not correlate independently with the mortality of COVID-19 patients (205). In contrast, age and clinical comorbidities were independently correlated with COVID-19-related death in other studies (121, 206). In living donor liver transplantation, ACE2 is a substitute marker for liver regeneration and is upregulated in liver tissue and serum (207). Therefore, during the early postsurgical stage, both liver transplant recipients and donors are more likely to develop SARS-CoV-2 infection because of their elevated ACE2 expression (208). Undiscovered SARS-CoV-2 infection of recipients can increase the risk of developing serious immunosuppression and postsurgical infection, which might cause multiple system organ injury or failure (186). Additionally, a donor with undiscovered SARS-CoV-2 infection may transfer the virus to recipients.

It is reported that using immunosuppressive medicines can modulate the inflammatory activity against SARS-CoV-2 infection (206), and the potential adverse effects need to be considered for liver transplant recipients as well (209). The application of early treatment may also serve as an essential step for the prevention of severe pneumonia in liver transplant recipients (210–212). It is recommended that patients with pre-existing liver disease rapidly receive antiviral treatment (207). According to EASL-ESCMID, special drugs recommended for the treatment of COVID-19 after liver transplantation include remdesivir, chloroquine/hydroxychloroquine with or without azithromycin, lopinavir/ritonavir, tocilizumab et al. (213, 214). Strict screening criteria for organ recipients and donors with coronavirus infection needs to be set to avoid further transmission (215).

### Effect of Coronaviruses on Alcoholic Liver Disease and Non-alcoholic Fatty Liver Disease

Patients with alcohol use disorder (AUD) or alcohol liver disease (ALD) are special components of the population with liver diseases (216). The COVID-19 pandemic has resulted in a social environment that leads people to drink at home. Selling of alcohol has increased by 55% in the week ending March 21 compared with the same time last year (217). A Chinese initial report has showed an over 2-fold increase in harmful drinking during the COVID-19 pandemic (218). Same effect was also seen in the USA, in which AUD and ALD is responsible for the highest hospitalization-cost among all chronic liver diseases. ALD patients usually have underlying medical conditions which can lead to higher risks of severe SARS-CoV-2 infection, including obesity with metabolic syndromes, chronic kidney diseases, and corticosteroid treatment for alcoholic hepatitis (219). Actually, patients with severe alcoholic hepatitis should not be treated with standard corticosteroid, especially in localities that are mostly affected by COVID-19 pandemic.

NAFLD is a chronic dysmetabolic disease which has become the most common liver disease in the world, with a prevalence rate of 30% in the western world (220). In addition, NAFLD is not isolated, it is often related to a series of risk factors, metabolic syndromes, and other diseases. The risk of severe SARS-CoV-2 infection also increases by the comorbidity of NAFLD (221). According to a report on 202 COVID-19 patients and their NAFLD status, COVID-19 progression was associated with male sex, age >60, higher BMI, and NAFLD (222). This study also indicated that NAFLD is an independent risk factor for COVID-19 progression (OR 6.4; 95% CI 1.5–31.2). NAFLD is also related to higher risk of abnormal hepatic function and longer clearance time of viruses. In another research, the moderate or high Fibrosis 4 (FIB-4) score can significantly and independently increase the risk of severe COVID-19 progression (223). Therefore, patients with NAFLD show a distinct risk as their metabolic dysfunction and underlying hepatic disorder.

## Effects of Anti-Coronavirus Treatments on the Liver

### Remdesivir

Remdesivir is an antiviral drug which is undergoing clinical trials for treating SARS-CoV-2 infection (224, 225). It was first used for treating Ebola virus infection with clinical experiments still on (226, 227). Results from ongoing experiments *in vitro* and *in vivo* demonstrated the activity of remdesivir against Paramyxoviruses, Filoviruses, and Coronaviruses (80). One study reported adverse events in three patients after using remdesivir, including nausea, vomiting, gastroparesis, and rectal bleeding (153). They also presented increased ALT and AST levels at 1–5 days after receiving the drug (228). However, it remains unclear whether this biochemical change was due to remdesivir or the virus because a large percentage of severely COVID-19 cases develop hepatic dysfunction. At present, there are insufficient data to give a definite adverse effect profile for remdesivir. Conclusive evidence of its effectiveness and adverse effects and calls for further clinical trials (229).

### Lopinavir/Ritonavir

Lopinavir and ritonavir, inhibitors of the HIV protease, are two HIV-1 drugs approved by the FDA (230, 231). Recently studies found that the antiprotease activity of these two drugs seem to be effective to against the SARS-CoV-2 (232). The adverse effects observed in ICU patients involved pancreatitis, hepatitis, liver decompensation, prolonged PR intervals and congenital QTc prolongation (233). Previous studies found that the serum amylase and hepatic enzymes were elevated in SARS patients using lopinavir/ritonavir (81). A recent study indicated that CYP3A4 metabolic pathways played essential roles in ritonavir-mediated hepatotoxicity (234). CYP3A participates in the generation of electrophilic content and oxygen free radicals, which covalently bind to macromolecular substances within hepatocytes, causing membrane lipid peroxidation and destruction of membrane integrity (210). Lopainavir/Ritonavir can also act on Ca^2+^-ATPase on the cell membrane, disrupt the balance between internal and external Ca^2+^ concentrations and dysregulate the biofunction of key organelles (including mitochondria and ER), resulting in injury or even necrosis of hepatocytes (233). Furthermore, overdose of lopinavir/ritonavir can stimulate ER stress pathways in the liver, inducing liver necrosis and inhibiting hepatocyte proliferation (233), and it also initiates inflammatory responses and worsens liver injury by aggravating oxidative stress (235). Several clinical experiments proved that the combined use of lopinavir/ritonavir with other drugs is effective in patients with COVID-19 (183, 232, 236). However, a study reported that the usage of lopinavir/ritonavir combined with arbidol cannot efficiently promote clearance of SARS-CoV-2 in patients (237). Administration of lopinavir and ritonavir seems to only be beneficial for patients who are at the early-stage of SARS-CoV and MERS-CoV infection (182, 238). Fan et al. found that a high percentage of patients exhibited abnormal levels of hepatic enzymes (57.8%) after receiving lopinavir/ritonavir compared with patients with normal liver function (31.3%) (39).

### Interferons

Interferon (IFN) is a type of endogenous signaling molecules secreted by host cells during the immune response to pathogen (239). Increased IFN levels activate the immune system to clear pathogens and suppress pathogen replication (106). There are two subclasses of IFNs which participate in the immune responses: IFN-a and IFN-b. IFN-a initiates effective host-mediated immune activity, which has shown value in the treatment of viral infections (including HBV and HCV) and cancers (240). IFN-b was originally used to treat the autoimmune multiple sclerosis (241). Non-specific immune-mediated reactions may be promising for other viral diseases, including SARS-CoV-2 (107, 111). Nevertheless, patients receiving IFN can also generate neutralizing antibody which decreases the efficiency of viral elimination (242). The adverse effects included leukopenia, lymphopenia, autoimmune hepatitis, and thyroid disease (154, 243).

### Baricitinib

Baricitinib is a JAK-STAT inhibitor for the treatment of rheumatoid arthritis patients who should not take more than one TNF antagonist (244, 245). Barcitinib was proven to affect the hyperinflammatory status which happened during SARS-CoV-2 infection and might avoid endocytosis and viral infection by depressing AAK1 activity (246, 247). Scientists should pay attention to the increasing number of reports on infections and thrombosis after using JAK inhibitors for the treatment of COVID-19 (248, 249). We should also evaluate adverse hepatic effects, particularly liver injury, cholestasis and hepatitis, which unexpectedly developed in a non-negligible number of cases (155). To our knowledge, this is the first strong evidence for a potential correlation between baricitinib and drug-induced liver injury, which is a rare and unpredictable adverse effect requiring case-by-case evaluation to exclude other possible reasons for the injury, including the application of drugs with recognized effects of drug-induced liver injury (250, 251).

### Tocilizumab

Tocilizumab is a monoclonal antibody against IL-6 receptors, which is originally used for treating rheumatoid arthritis (252, 253). As study from a medical institution in Wuhan reported that 20 severe COVID-19 cases all exhibited rapid decrease in fever after adding tocilizumab to lopinavir, methylprednisolone, and oxygen therapy, increased the oxygenation efficacy to 5% and the hospital discharge rate to 95% (254). Further studies are undergoing for evaluating the efficacy of combining tocilizumab with other antivirals drugs (82, 255). Tocilizumab can lead to moderate elevation in serum aminotransferase, which is usually short-lived and asymptomatic, but it is also correlated with jaundice and occasional reactivation of HBV (256, 257). Tocilizumab need to be withheld when the serum neutrophil is lower than 1,000 cells/mm^2^, platelet is lower than 100,000 cells/mm^2^, and/or hepatic enzymes are higher than three times of the upper normal limit (258).

## Effects of Coronavirus Vaccines on the Liver

Vaccines against SARS-CoV-2 will be vital for avoiding the spread of the virus and alleviating social panic, but multiple aspects should be considered to prevent an activated innate inflammatory response, increased incidence of autoimmune diseases, and vaccine-induced liver injury (259). In general, the development of vaccinations is costly, and it usually takes long time to finish strict animal and clinical trials before approval for public applications (260). However, under the situation of the COVID-19 outbreak, the medical community is facing tremendous pressure to rapidly develop effective vaccines (261). In past pandemics such as those involving Ebola, H1N1, SARS, and MERS, vaccine development was unable to be completed owing to the end of the pandemic and the reallocation of scientific funds (262–265). Since July 2^nd^, 2020, there has been 158 vaccine candidates for COVID-19, 135 of which are in the preclinical or the developing stage. Until now, mRNA-1273 (266), Ad5-nCoV (267), INO-4800 (268), LV-SMENP-DC (269), Pathogen-specific aAPC (270), and ChAdOx1 (271) have entered the phase II/III clinical trials (Table 5).

**Table 5:** The efficacy and current status of COVID vaccines.

**Vaccine**	**Platform**	**Efficacy**	**Safety**	**Stage of development**
BNT162b2 BioNTech/Fosun Pharma/Pfizer	3 LNP-formulation encapsulated mRNA	95%	Contraindicated if there is a history of severe or immediate allergy to any component of the vaccine	FDA EUA
mRNA-1273 Moderna/NIAID	Prefusion stabilized S protein mRNA encapsulated in LNP	94.1%	Contraindicated if there is a history of severe or immediate allergy to any component of the vaccine	FDA EUA
AZD1222 ChAdOx1nCoV-19/University of Oxford/AstraZeneca	Chimpanzee adenovirus vector displaying Spike protein on its surface	70.4%	Cases of transverse myelitis have been reported	Phase 3 clinical trial ISRCTN89951424 NCT04516746 NCT04540393 CTRI/2020/08/027170
Ad5-nCoV CanSino Biological Inc	Adenovirus serotype 5 expressing Spike protein	96%	Defective vector replication	Phase 3 clinical trial NCT04526990 NCT04540419
Ad26 CoV S1 Janssen Pharmaceutical	Adenovirus serotype 26 expressing Spike protein	72%	Low Seroprevalence of antibodies	Phase 3 clinical trial NCT04505722 NCT04614948
NCX-CoV2373 Novavax	Full length recombinant SARS-CoV-2 glycoprotein nanoparticles adjuvanted with Matrix M	89.3%	Adjuvant of M-matrix may be allergenic	Phase 3 clinical trial NCT04533399
CoronaVac Sinovac	Formalin inactivating whole virus particles + alum adjuvant	50.4%	Inactivated SARS CoV-2 with alum hydeoxide adjuvant	Phase 3 clinical trial NCT04456595 NCT04582344 NCT04617483
BBIBP-CorV Sinopharm Wuhan Institute of Biological Products/Beijing Institute of Biological Products	Inactivated SARS-CoV-2	100%	Inactivated whole virion SARS-CoV-2	Phase 3 clinical trial NCT04612972

Under a pandemic situation, vaccines with the greatest potential to treat COVID-19 are protein sub-unit vaccines, viral vectored vaccines, and RNA- or DNA-based vaccines (272, 273). Plasmid DNA and mRNA vaccines have attracted scientists' attention and effort as they might be applicated to prophylaxis and therapy for personalized treatment and social health solutions (274). These two vaccines can be rapidly and directly produced from the sequence of the targeted protein by general manufacturing methods, either human or virus in origin (275). For vaccinations, constructing a genetic sequence for the antigen rather than deactivating the pathogen or constructing a recombined protein is simpler and quicker and reduces the potential risks of working with live pathogens (276).

These vaccines do not require culture in the lab; they reduce the risk of exposure to live viruses and encode targeted antigens without generating other toxins, but this does not mean that they do not have risks (277, 278). The pitfall of possibly effective adjuvant inflammation is the potential hepatotoxicity of RNA- or DNA-based vaccines (276). As mentioned above, antivirals and anticancer drugs that contain engineered nucleoside analogs can be toxic (138–140), and such toxicity cannot be predicted by preclinical trials and safety tests due to species difference between human and animal (260). The clinical adverse effects include myopathy, acute pancreatitis, lipodystrophy, hepatic steatosis, and neural injury (273, 274). Vaccine hepatotoxicity was found in preclinical studies with a potential mRNA target obtained from lipid nanoparticles for Crigler-Najjar syndrome, being chosen because only a small dose of protein is required (279). The expression of the mRNA is believed to play a potential role in hepatotoxicity, and repeat dosages were applied (280). In a clinical trial for the mRNA rabies vaccine, self-limited adverse effects reflected by innate immune activities were discovered, even though the authors stated that the vaccine was generally safe (278). However, adverse events to this extent are not observed when using DNA vaccines (272, 275). The double-stranded structure of the DNA plasmid is regarded as a substance that stimulates the immune system through non-TLR pathways (274). Indeed, plasmid DNA also acts on the TBK1-Sting pathways (275), leading to the secretion of IFN-1, which serves as an adjuvant for the initiation of inflammatory responses against antigens (281). It should also be noted that for monoclonal antibodies, repeat administration of mRNA would likely be required, which may increase the efficacy as well as the risk of toxicity (276). Thus, finding the balance of inflammation and deleterious toxicity by controlling adjuvant activities of mRNA remains a work in progress.

Chronic liver disease (CLD) is a contraindication to multiple COVID-19 vaccines, such as: Pfizer Biontech vaccine (Bnt 162B2) (282), Moderna vaccine (mRNA-1273) (283), Chadox1 nCoV-19 vaccine (AZD1222) (284), etc. As a result, there is little data on COVID-19 vaccination in patients with CLD. Given the reduced immunogenicity of non-coronavirus vaccines in patients with CLD, it is unclear whether the COVID-19 vaccine will produce an adequate and durable immune response to the virus as in healthy people (285). The role of increased liver disease severity in determining the immune response to COVID-19 vaccine is unclear. Although no significant hepatotoxicity was reported in the trials conducted, the number of registered patients with liver disease was too small to draw definitive conclusions about the safety of the vaccine in this population. Many clinical trials in patients with liver disease are currently under way worldwide. In view of CLD patients at higher risk of COVID-19-related death, EASL and AASLD recommended that priority for COVID-19 vaccination should be given to patients with advanced liver disease and those who have undergone liver transplantation for more than 3 months (286, 287). Patients with chronic liver disease who are taking antivirals or immunosuppressive drugs should not stop taking drugs before and after vaccination.

## Discussion

The recent COVID-19 pandemic has become a threat to global health, and the virus is still evolving. Lessons from previous outbreaks of coronaviruses and influenza epidemics suggest that viral infections can lead to severe respiratory syndromes and corresponding complications (such as abnormal liver function, cardiac insufficiency and renal failure) as a result of the combination of systemic and partial inflammatory responses. As the most important metabolic organ in the human body, the liver is dramatically affected by coronavirus infection. On the other hand, pre-existing liver diseases also influence the severity and motility of patients with coronavirus infection.

SARS-CoV, MERS-CoV, and SARS-CoV-2 are three coronaviruses with remarkable genetic similarity and can all cause acute respiratory inflammation in humans. Compared with MERS-CoV, SARS-CoV, and SARS-CoV-2 share greater similarity, as both attach to host cells using the ACE2 receptor; in contrast, MERS-CoV binds to DPP4 of the host cells. Nonetheless, the manifestations of liver injury are rather the same among them, characterized by decreased albumin and elevation in ALT, AST, liver enzymes and bilirubin. Increases in GGT and ALP are also observed in COVID-19 patients, suggesting injury of bile duct cells in the liver. Thus, liver injury might also be the result of bile duct cell injury, as liver biopsies obtained from a few COVID-19 patients did not show viral inclusions but rather microvesicular steatosis. The pathological changes that occur are also similar among the three coronaviruses, commonly manifesting as microvascular steatosis and moderate lobular and portal inflammation. Based on previous studies, the frequency and extent of liver injury in severe cases with coronavirus infection were remarkably higher than those in mild cases. As a result, we conclude that these three coronaviruses can cause liver injury that results in similar manifestations and that the degree of liver injury correlates positively with the severity of infection.

Although numerous clinical studies have indicated a strong correlation between liver injury and coronaviruses, the mechanism by which coronaviruses damage hepatocytes and affect hepatic function is still unclear. Several pathophysiological theories have been proposed. First, ACE2-mediated hepatocyte damage is known to be the most direct effect of SARS-CoV-2 infection on the liver. Upregulation of ACE2 in hepatocytes facilitates the invasion of SARS-CoV-2 and causes greater virus virulence in the liver. Considering the important role of ACE2 in coronavirus infection, hrsACE2 may become a promising therapy for patients with SARS-CoV or SARS-CoV-2 infection. Second, immune activity is largely enhanced during coronavirus infection. Once infected with coronaviruses, a large number of cytokines (IL-6, IL8, IFN-γ, and TNF-α, etc.) are secreted by immune cells and released into the blood, inducing inflammation in various tissues or even ARDS, SIRS and MOF. This suggests that immunotherapy is essential for patients with coronavirus infection, and accordingly, interferon-α and corticosteroids are widely used owing to their anti-inflammatory function. However, as immune dysfunction leads to serious consequences, close monitoring of serum cytokines is necessary during immunotherapy. Third, hypoxia can cause persistent elevation in reactive oxygen species, which can promote the secretion of various pro-inflammatory substances that induce liver injury. Therefore, monitoring hypercoagulable states in patients, including thrombocytopenia and increased D-dimer and ALP levels, will be meaningful for preventing thrombosis and further ischemia and hypoxia. To summarize, all of these factors can affect hepatic function and cause liver damage during the course of coronavirus infection. As liver injury is due to multiple factors, more attention should be paid to the pathogenic mechanisms involved, not only in laboratory experiments but also in clinical monitoring and follow-up visits.

The acquisition and clearance of coronavirus infection is largely dependent on the health condition of the patient as well as and any pre-existing diseases. Hepatitis B and C, liver cirrhosis, liver cancer, and immunosuppressive drugs after liver transplantation generally lead to an immunocompromised state. As the numbers of infected individuals and clinical studies of SARS-CoV-2 are much greater than those of SARS-CoV or MERS-CoV, the correlation between pre-existing liver disease and COVID-19 is clearer and more convincing than that for SARS or MERS. Due to delayed SARS-CoV-2 clearance in those with HBV infection, the severity and mortality rate is higher in patients with HBV infection than in those with HBV negativity. For those who have already developed liver cirrhosis, the Child-Pugh scores are likely to increase because of liver injury caused by COVID-19. Moreover, complications of COVID-19 occur earlier and to a larger extent in patients with systemic immunocompromised status. COVID-19 also largely influences the treatment of liver diseases. For hepatitis B/C patients undergoing anti-HBV treatment, discontinuation of high-dose corticosteroid therapy might cause reactivation of HBV during SARS-CoV-2 infection. Furthermore, lopinavir and ritonavir have been proven to increase the chance of developing liver injury in patients with HBV or HCV infection. To prevent the risk of virus transmission, certain examinations such as endoscopy and vascular radiography are being restricted to only severe emergencies (for example, internal bleeding). Although many widely recognized institutions (including ASCO, ESMO, ILCA, EASL, and AASLD) have provided guidance for liver disease treatment in the presence of SARS-CoV-2 infection, more optimized treatments need to be explored to accomplish the lowest risk of disease deterioration and complications.

At present, drugs that are used for treating coronavirus infection include remdesivir, lopinavir/ritonavir, interferon-a, baricitinib, tocilizumab, ACE2 inhibitors, and hrsACE2. Unfortunately, no drug has been proven to be absolute effective for coronavirus therapy. In fact, there are still no excellent drugs for therapy of SARS and MERS, which emerged many years ago. The difficulty in finding optimized drugs for coronavirus infection is mainly due to severe adverse effects. Remdesivir, lopinavir, and ritonavir have all been reported to increase the probability of liver injury, and the extent of liver injury is closely related to the dose of these drugs. IFNs have the potential to initiate a non-specific immune response, causing hepatocyte damage and autoimmune hepatitis and increasing the risk of developing severe complications such as ARDS and SIRS. As a JAK inhibitor, baricitinib can increase the risk of thrombosis and further lead to liver injury. Tocilizumab can also reactivate HBV in SARS-CoV-2 coinfection, which will delay the recovery of both viral hepatitis and COVID-19. ACE inhibitors and hrsACE2 might impede invasion of coronaviruses and also attachment to host cells in various tissues (such as the lung, liver, gastrointestinal tract and kidney), preventing organ damage. Overall, vaccines against coronaviruses will be vital in preventing their outbreaks, but multiple factors need to be considered to avoid an activated innate inflammatory response, an increased incidence of autoimmune diseases, and vaccine-induced liver injury. Although vaccines with the greatest potential are RNA- or DNA-based vaccines, their positive and adverse effects are seldomly detected due to species differences between humans and lab animals. Therefore, the development of vaccinations usually takes a long time to carry out strict animal and clinical trials before being approved for public applications, which is a challenging task for both society and scientists.

Here, we discussed the potential effects of three coronaviruses and their related treatments on the liver, yet there remains a huge lack of clinical and laboratory experiments to provide strong evidence. The reasons can be summarized as follows:

1) The clinical data of patients have not been fully explored. As the main manifestations of coronavirus infections are respiratory inflammation and damage, most studies have focused on impacts on the lung. However, coronavirus infections can also cause severe complications in other organs, such as the kidney and liver. Therefore, close monitoring of hepatic biochemical parameters is essential to reduce the deterioration of liver disease and death from liver failure. In general, other known factors with hepatotoxicity must be excluded when evaluating the significance of a factor on liver injury.
2) Some studies are waiting for follow-up data for COVID-19 patients, as well as data from COVID-19 patients with long course of disease. Exploration on the relationship between coronavirus infections and chronic liver diseases, such as liver cirrhosis, liver cancers and liver transplantation, is of great significance, and long-term data collection is needed. A systematic record of clinical information and liver diseases, including biochemical indicators, virus-related indicators, clinical symptoms, immunity states and psychological states of each stage of the disease. We should also utilize information on family history to deeply illustrate the relationship between pre-existing liver disease and COVID-19-induced liver injury.
3) Genetic regulatory mechanisms of coronavirus infections warrant further exploration. For example, the level of ACE2 expression in hepatocytes can largely decide the extent of direct liver injury, but upregulation of ACE2 expression does not occur in all patients. Therefore, genetic variations and transcriptome data obtained from second-generation sequencing can provide more ideas regarding the mechanism of COVID-19 susceptibility and its complications.
4) Suitable animal models are urgently needed. For studying the relationship between pre-existing liver diseases and COVID-19-induced liver injury, it is better to establish an animal model that has both liver diseases and COVID-19. The safety and potential adverse events of drugs and vaccines that might occur in COVID-19 patients should be evaluated in animal experiments.

## Conclusion

Our understanding of coronaviruses, their diagnosis, treatment, and prevention is rapidly evolving. As the pandemic spreads and new evidence is published, it is important to study the effect of coronaviruses on the liver and identify the risk factors for hepatic complications in patients with coronavirus infection. There is an urgent need to develop a clinical guidance for liver diseases patients with coronavirus infection. A complete record of patients with coronaviruses infection with systematic recording of clinical information and liver diseases will be useful to the identification of hepatic complications, the development of hepatic complications risk models, and the prediction of response to treatment.

## Author Contributions

XW and JL conducted literature retrieval and data collection. XW summed up the information and wrote the first draft. LY and ZL took part in revising the article critically and made substantial contributions to the conception and design. All authors approval for the version for publication and agree to be accountable for all aspects of the work.

## Conflict of Interest

The authors declare that the research was conducted in the absence of any commercial or financial relationships that could be construed as a potential conflict of interest.

## Publisher's Note

All claims expressed in this article are solely those of the authors and do not necessarily represent those of their affiliated organizations, or those of the publisher, the editors and the reviewers. Any product that may be evaluated in this article, or claim that may be made by its manufacturer, is not guaranteed or endorsed by the publisher.

## Abbreviations

SARS-CoV: severe acute respiratory syndrome coronavirus
MERS-CoV: Middle East respiratory syndrome coronavirus
COVID-19: coronavirus disease 2019
SARS-CoV-2: severe acute respiratory syndrome coronavirus 2
ACE2: angiotensin-converting enzyme 2
ARDS: acute respiratory distress syndrome
MOF: multiple organ failure
S proteins: spike proteins
ER: endoplasmic reticulum
Dpp-4: dipeptidyl-peptidase 4
ICU: intensive care unit
CCU: critical care unit
ALT: glutamic-pyruvic transaminase
AST: glutamic-oxalacetic transaminease
GGT: gamma-glutamyl transferase
ALP: alkaline phosphatase
Ang II: angiotensin II
hDpp-4: human DPP-4
hrsACE2: human recombinant soluble ACE2
TNF: tumor necrosis factor
IL-6: interleukin-6
SIRS: systemic inflammatory reaction syndrome
HIRI: Hepatic ischemia–reperfusion injury (HIRI)
ROC: reactive oxygen species (ROS)
ALD: alcoholic liver disease
NAFLD: non-alcoholic fatty liver disease
ASCO: American Society of Clinical Oncology
ESMO: the European Society for Medical Oncology
ILCA: the International Liver Cancer Association
EASL: the European Association for the Study of the Liver
ASSLD: the American Association for the Study of Liver Diseases
IFNs: Interferons.
